# Lipid-Induced Epigenomic Changes in Human Macrophages Identify a Coronary Artery Disease-Associated Variant that Regulates *PPAP2B* Expression through Altered C/EBP-Beta Binding

**DOI:** 10.1371/journal.pgen.1005061

**Published:** 2015-04-02

**Authors:** Michael E. Reschen, Kyle J. Gaulton, Da Lin, Elizabeth J. Soilleux, Andrew J. Morris, Susan S. Smyth, Christopher A. O'Callaghan

**Affiliations:** 1 Wellcome Trust Centre for Human Genetics, University of Oxford, Oxford, United Kingdom; 2 Nuffield Department of Clinical Laboratory Sciences, University of Oxford and Department of Cellular Pathology, John Radcliffe Hospital, Oxford, United Kingdom; 3 Division of Cardiovascular Medicine, The Gill Heart Institute, University of Kentucky, Lexington, Kentucky, United States of America; 4 Department of Veterans Affairs Medical Center, Lexington, Kentucky, United States of America; European Institute of Oncology, ITALY

## Abstract

Genome-wide association studies (GWAS) have identified over 40 loci that affect risk of coronary artery disease (CAD) and the causal mechanisms at the majority of loci are unknown. Recent studies have suggested that many causal GWAS variants influence disease through altered transcriptional regulation in disease-relevant cell types. We explored changes in transcriptional regulation during a key pathophysiological event in CAD, the environmental lipid-induced transformation of macrophages to lipid-laden foam cells. We used a combination of open chromatin mapping with formaldehyde-assisted isolation of regulatory elements (FAIRE-seq) and enhancer and transcription factor mapping using chromatin immuno-precipitation (ChIP-seq) in primary human macrophages before and after exposure to atherogenic oxidized low-density lipoprotein (oxLDL), with resultant foam cell formation. OxLDL-induced foam cell formation was associated with changes in a subset of open chromatin and active enhancer sites that strongly correlated with expression changes of nearby genes. OxLDL-regulated enhancers were enriched for several transcription factors including C/EBP-beta, which has no previously documented role in foam cell formation. OxLDL exposure up-regulated C/EBP-beta expression and increased genomic binding events, most prominently around genes involved in inflammatory response pathways. Variants at CAD-associated loci were significantly and specifically enriched in the subset of chromatin sites altered by oxLDL exposure, including rs72664324 in an oxLDL-induced enhancer at the *PPAP2B* locus. OxLDL increased C/EBP beta binding to this site and C/EBP beta binding and enhancer activity were stronger with the protective A allele of rs72664324. In addition, expression of the *PPAP2B* protein product LPP3 was present in foam cells in human atherosclerotic plaques and oxLDL exposure up-regulated LPP3 in macrophages resulting in increased degradation of pro-inflammatory mediators. Our results demonstrate a genetic mechanism contributing to CAD risk at the *PPAP2B* locus and highlight the value of studying epigenetic changes in disease processes involving pathogenic environmental stimuli.

## Author Summary

Coronary artery disease is a complex disease where over 40 genomic loci contributing to genetic risk have been identified. However, identifying the precise variants, genomic elements and genes that mediate this risk at each locus has proved challenging. We hypothesized that some genetic risk variants may influence a key step in development of coronary artery disease, which occurs when macrophages encounter environmentally-derived lipid. These cells take up lipid and accumulate in atherosclerotic plaques in the walls of blood vessels where they contribute to the inflammatory atherosclerotic disease process. Therefore, we studied the effects of this lipid exposure on the genomic activity of these cells. Environmental lipid exposure triggered changes in transcriptional regulation and gene expression. Variants at coronary artery disease risk loci were enriched for genomic regions altered by lipid exposure. We studied one such risk variant rs72664324 in detail and found that it altered binding of the C/EBP-beta transcription factor and altered expression of the *PPAP2B* gene. *PPAP2B* encodes an enzyme that degrades pro-inflammatory substances. Our study demonstrates a hitherto unknown genetic mechanism underlying atherosclerotic heart disease and demonstrates the value of studying changes in transcriptional regulation in key disease processes involving environmental influences.

## Introduction

Coronary artery disease (CAD) is the leading cause of death worldwide [1]. Most cases are caused by atherosclerosis, a form of chronic inflammation in arterial walls that involves the accumulation of lipid-containing plaques [2,3]. Blood levels of low-density lipoproteins (LDL) are a major environmentally-influenced risk factor for CAD and one of the more successful preventative treatments, statin therapy, lowers LDL levels [4–6]. A greater understanding of the molecular events by which lipoproteins cause atherosclerosis is a prerequisite for rational development of new therapies targeting this aspect of atherosclerosis.

CAD is a complex disease with a strong heritable component [7]. A major development in the study of the heritable component of complex diseases has been the application of genome-wide association studies (GWAS) to identify regions of the genome that contain genetic variants that mediate this heritable risk. These studies have identified over 40 genomic loci harboring genetic variants that influence CAD risk [8]. Although the contribution of each locus to the overall risk is typically small, the gene pathways mediating risk at each individual locus contain useful biological information and may involve potential therapeutic targets [8]. The majority of CAD risk variants do not alter the sequence of protein coding genes; thus the mechanism by which a risk locus operates is not typically identified by GWAS studies themselves. Furthermore, the particular SNP identified by a GWAS study may only be in linkage disequilibrium (LD) with the causative SNP. Several studies have identified CAD-associated variants that alter transcription factor binding or miRNA binding, suggesting that many causal variants underlying CAD risk influence gene regulatory processes [9–11]. However, for most CAD loci the mechanism by which CAD-associated variants affect the disease is not known. Indeed for most CAD loci, it is not clear in which cell type the risk variants exert their effects.

In atherosclerosis, LDL cholesterol is deposited in the arterial wall and undergoes modifications, such as oxidation, that in turn promote pro-inflammatory processes [2,12]. Monocytes are recruited to these sites and differentiate into macrophages, which have a variety of scavenger surface receptors for oxidized LDL (oxLDL) [2,12]. The uptake of oxLDL by macrophages occurs via scavenger receptors, phagocytosis and macro-pinocytosis and is fundamental to the development of atherosclerotic lesions [12]. An imbalance in the uptake and degradative metabolism of oxLDL leads to the accumulation of lipid-laden vesicles in macrophages giving rise to a foam cell phenotype [2,12]. A central role for macrophages in atherosclerosis is evidenced by the inhibition of atherosclerosis in mice with severe macrophage deficiency due to knockout of macrophage colony-stimulating factor [13].

Foam cells contribute to the pathogenesis of atherosclerosis by releasing pro-inflammatory mediators that recruit additional cells and matrix metallo-proteinases that can destabilize plaques [2,12]. In addition, foam cell apoptosis causes release of toxic lipids into the necrotic core of the lesion [14,15]. Plaque rupture can expose this thrombogenic mixture to luminal blood leading to thrombosis and blood vessel occlusion [2]. Several studies have documented changes in lipid response and oxidative stress response pathways during foam cell formation, but understanding of how oxLDL influences transcriptional regulation in macrophages is incomplete [16,17]. Nevertheless, reprogramming of the macrophage response to lipid is a plausible therapeutic strategy.

Development and maintenance of cell type identity depends on the binding of hundreds of different transcription factors to thousands of *cis*-regulatory elements [18]. Active *cis*-regulatory regions are typically characterized by nucleosome depletion, a necessary condition for DNA binding by many transcription factors [19–21]. A variety of techniques have emerged which allow mapping of this accessible open chromatin, such as DNAseI hypersensitivity or formaldehyde-assisted isolation of regulatory elements (FAIRE-seq) [22,23]. In parallel, chromatin immunoprecipitation (ChIP-seq) approaches have been developed to identify genomic sites where histones in nucleosomes that flank open chromatin have undergone post-translation modifications that indicate the function, such as enhancer function, of the open chromatin [18,24]. The identification of cis-regulatory elements is useful in the study of functional genetic risk variants. Overlap of a risk variant with a functional regulatory element in a particular cell type indicates that the variant might exert its effect in that cell type by altering the activity of the regulatory element [23,25,26]. Several large-scale studies have demonstrated that transcriptional regulatory sites identified via high-throughput assay in specific human cell types are enriched for disease-associated GWAS variants, highlighting the importance of pinpointing cell type regulatory elements [27,28].

In this study we employ FAIRE-seq and ChIP-seq assays to, first, show that exposure of primary human macrophages to oxLDL causes changes in chromatin accessibility and histone modification at a subset of regulatory sites. We then demonstrate that these changes are correlated with local expression of genes involved in foam cell and atherosclerotic processes and also binding of key transcription factors such as C/EBP-beta. We also show that these sites preferentially harbor variants influencing CAD risk and identify a regulatory variant rs72664324 that affects *PPAP2B* expression through altered C/EBP-beta binding and enhancer activity. Finally, we demonstrate that the protein product of PPAP2B (Lipid Phosphate Phosphohydrolase 3LPP3) is upregulated in foam cells and that this upregulation is associated with increased LPP3 enzymatic activity for degrading pro-inflammatory mediators. Our approach of studying chromatin profiles in the context of a major environmental disease stimulus as a means to identify the causal mechanisms of GWAS loci is widely applicable to other diseases.

## Results

### Oxidized LDL triggers changes in expression of genes near CAD risk variants

**Fig 1:**
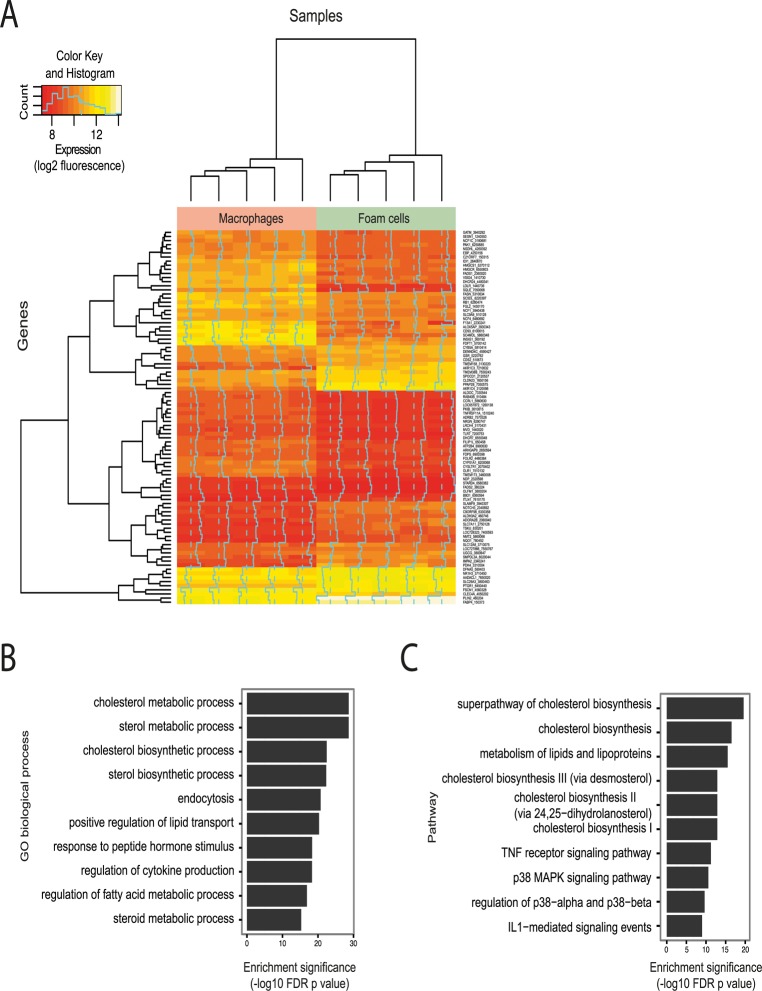
Effect of oxLDL-induced foam cell formation on gene expression in human macrophages. (**A**) Heatmap showing genes differentially expressed between macrophages and foam cells (subset with adjusted p < 0.0001). Each column in each half of the main panel represents one of the 5 donors. Each row in the figure represents one gene. Most changes are common to all donors. Dendrograms indicate clustering of genes by expression profile (left axis) and by sample (top axis). Dashed vertical lines indicate the median expression signal and solid vertical lines indicate expression for that gene and sample relative to the median. The inset indicates the expression distribution for the data and the color key for the heatmap. (**B and C**) Enrichment of differentially expressed genes for gene sets related to biological processes (**B**) and biological pathways (**C**).

Exposure of primary human macrophages to oxLDL resulted in marked lipid uptake and transformation to a lipid-laden foam cell phenotype, as demonstrated by oil red O lipid staining (S1 Fig). We measured gene expression levels before and after exposure to oxLDL and identified 1,283 and 1,376 genes significantly up- and down-regulated respectively (S1A/B Table, Fig. 1A). Among the most differentially expressed genes were several with previously known involvement in foam cell formation, including the low-density lipoprotein receptor (*LDLR)* and perilipin 2 (*PLIN2)* [2,29] (Table 1). We observed enrichment among all differentially expressed genes for gene ontology terms pertaining to lipid and sterol handling (Fig. 1B). Pathway analyses on the differentially regulated genes further highlighted pathways involved in inflammation (Fig. 1C).

**Table 1:** Top 10 most up-regulated and down-regulated genes during oxLDL-induced foam cell formation.

	Up-regulated genes			Down-regulated genes		
Rank	Gene symbol	Gene	Fold change (log2)	Gene symbol	Gene	Fold change (log2)
1	*PLIN2*	Perilipin 2	2.70	*LDLR*	Low Density Lipoprotein Receptor	-3.09
2	*AKR1C4*	Aldo-Keto Reductase Family 1, Member C4	2.50	*F13A1*	Coagulation Factor XIII, A1 Polypeptide	-2.81
3	*AKR1C3*	Aldo-Keto Reductase Family 1, Member C3	2.45	*SC4MOL(MSMO1)*	Methylsterol Monooxygenase 1	-2.57
4	*ABCG1*	ATP-Binding Cassette, Sub-Family G (WHITE), Member 1	2.44	*SQLE*	Squalene Epoxidase	-2.46
5	*PDK4*	Pyruvate Dehydrogenase Kinase, Isozyme 4	1.95	*INSIG1*	Insulin Induced Gene 1	-2.15
6	*PPAP2B*	Phosphatidic Acid Phosphatase Type 2B	1.65	*FCGBP*	Fc Fragment Of IgG Binding Protein	-2.01
7	*LOC644496*	Pseudo gene similar to cathepsin L1 preproprotein	1.54	*CD93*	CD93 Molecule	-2.00
8	*TMEM158*	Transmembrane Protein 158	1.51	*ALOX5AP*	Arachidonate 5-Lipoxygenase-Activating Protein	-1.91
9	*FABP4*	Fatty Acid Binding Protein 4, Adipocyte	1.50	*DHCR7*	7-Dehydrocholesterol Reductase	-1.83
10	*SLC7A11*	Solute Carrier Family 7	1.45	*FADS1*	Fatty acid desaturase 1	-1.81

We next explored the relationship between gene expression changes during foam cell formation and CAD risk variants. For this we identified genomic intervals containing all SNPs in high linkage disequilibrium (LD) (r^2^ > 0.8) with known CAD-associated variants and then identified all genes within 50kb of these CAD risk locus intervals (S2 Table). These 132 genes at CAD risk loci were enriched for processes relevant to atherosclerosis, including lipid handling and foam cell formation (S3, S4 Tables). Exposure of macrophages to oxLDL altered the expression of 19 of these 132 genes, a significant excess compared to expectation across all expressed genes (Binomial p = 0.025). This suggests that CAD variants may act on nearby genes to influence the macrophage response to oxLDL, a key stage in the pathogenesis of atherosclerosis.

### OxLDL induces major changes in macrophage chromatin accessibility

**Fig 2:**
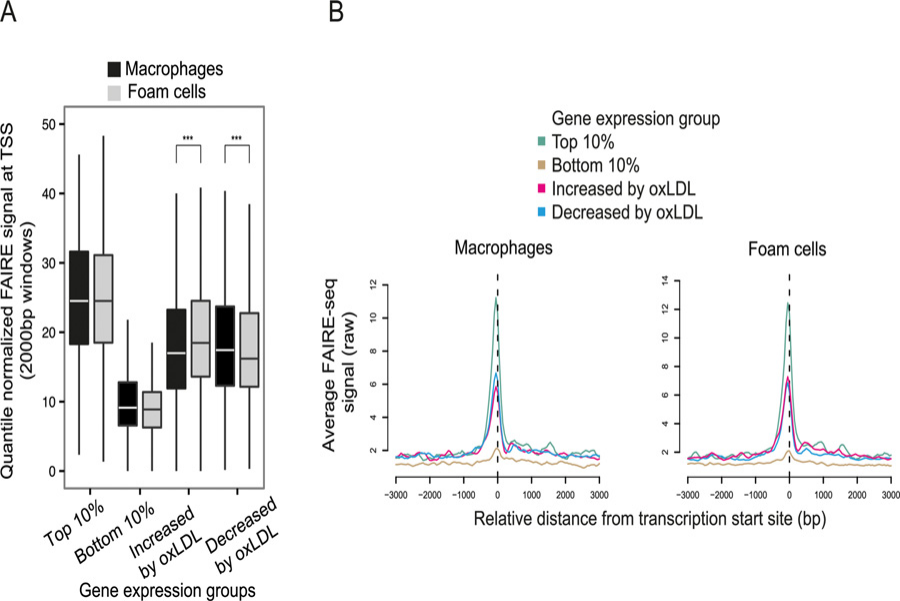
OxLDL-induced changes in chromatin structure determined by FAIRE-seq. (**A**) Normalized FAIRE-seq signal in 2000bp windows centered on the TSS for gene sets grouped by their expression characteristics. OxLDL-induced changes in open chromatin were associated with concordant changes in gene expression (higher FAIRE signal indicates more open chromatin, *** p < 0.0005). (**B**) Profile view of FAIRE-seq signal around TSSs showing sharply delineated promoter open chromatin profiles and their relationship with expression.

To understand the transcriptional pathways mediating the effect of oxLDL on gene expression, we identified candidate regulatory DNA elements that underwent oxLDL-induced changes in chromatin structure. We began by generating genome-wide maps of open chromatin in primary human macrophages before and after oxLDL exposure, with consequent foam cell formation. Formaldehyde-assisted isolation of regulatory elements with high-throughput sequencing (FAIRE-seq) identified 130,491 and 123,400 nucleosome-depleted open chromatin sites in macrophages and foam cells respectively (S5, S6 Tables) [19,22]. Sites at promoters, defined as ≤ 1kb upstream of the Refseq transcriptional start site (TSS), accounted for 6.1% of open chromatin sites in macrophages and 7.1% in foam cells (S2 Fig). The promoters of genes with a low level of expression had low FAIRE signal consistent with a relatively nucleosome-bound chromatin profile. By contrast, the promoters of maximally expressed genes had a high FAIRE signal consistent with nucleosome-depletion (Fig. 2A, 2B) [19]. Genes whose expression was altered by oxLDL exposure had an intermediate chromatin profile at their promoters. OxLDL significantly increased nucleosome depletion at the promoters of up-regulated genes, (p = 1.08 x 10^-27^) and reduced it at the promoters of down-regulated genes (p = 5.42 x 10^-21^) indicating its capacity to trigger chromatin remodelling (Fig. 2A, 2B).

**Fig 3:**
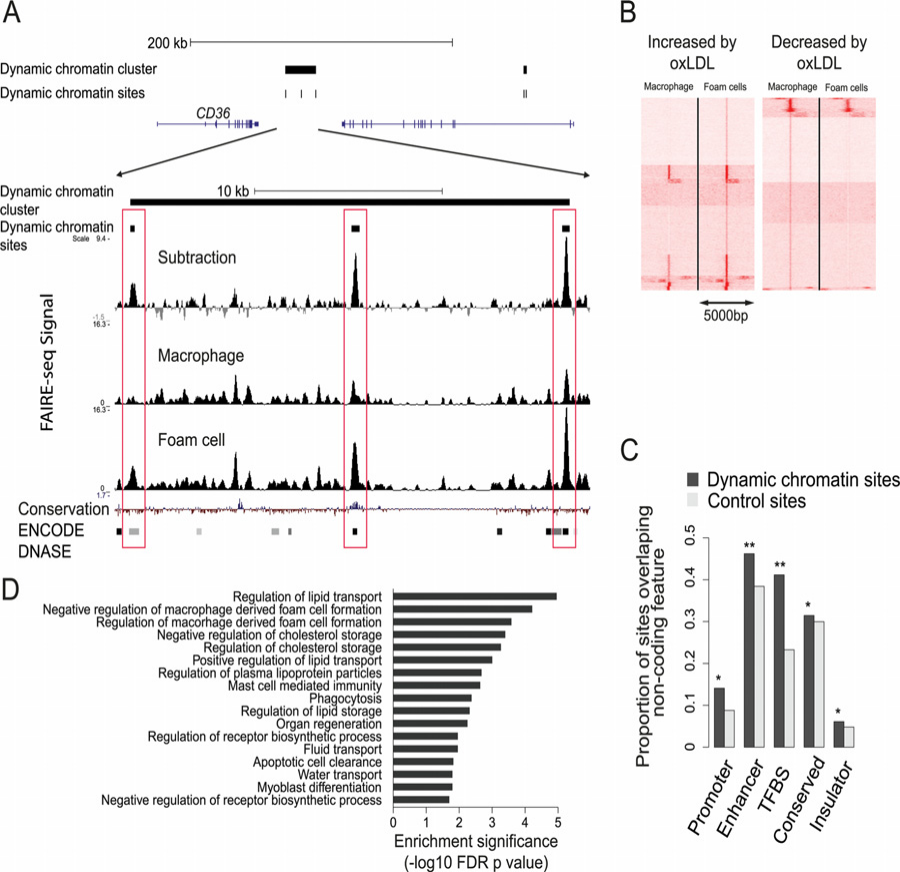
Characterization of the dynamic chromatin landscape resulting from oxLDL exposure. (**A**) Representative chromatin profile at the *CD36* locus showing a dynamic chromatin cluster comprising three dynamic chromatin sites; the lower panel close-up view shows the relevant FAIRE-seq signals for macrophages, foam cells and the normalized dynamic signal (foam cell minus macrophage signal). Dynamic sites are highlighted in red boxes and are known DNAse hypersensitivity sites in other cell types in the ENCODE database; one site shows conservation in vertebrates. (**B**) Heatmap of open chromatin profile at all dynamic chromatin sites, split into panels showing sites with increased or decreased FAIRE-seq signal. Each line represents a 5kb window centered on an individual site. Sites are clustered into groups with similar signal patterns. (**C**) Dynamic chromatin sites are enriched for non-coding regulatory elements found in 9 other cell types (* p < 0.05, ** p < 0.005, TFBS—transcription factor binding site). (**D**) Dynamic chromatin clusters are enriched for gene sets involved in foam cell formation and lipid handling.

All genomic sites with significant oxLDL-induced changes in chromatin profile were identified and termed dynamic chromatin sites (Fig. 3A). OxLDL changed the chromatin profile at around 10% of all open chromatin sites (13,516 sites of which 12,754 (94%) were non-promoter sites (defined as 1kb from a known RefSeq TSS)(S7 Table). 7,276 (54%) of these 13,516 sites had a more nucleosome-depleted profile and 6,240 had a more nucleosome-bound profile in foam cells (Fig. 3B). The size distribution of the dynamic chromatin sites demonstrates that changes at most non-promoter sites involve displacement of a single nucleosome, whereas those at promoter sites may involve displacement of one or two nucleosomes (S3 Fig). Overall, only a fraction of macrophage open chromatin sites, the majority distal to known promoter regions, are changed in response to oxLDL.

We next determined whether dynamic chromatin sites had characteristics representative of true regulatory elements. First, we tested the extent to which these sites overlapped regulatory elements identified in the ENCODE project, compared to control DNA sequences randomly chosen from within 10kb of each site (see Methods). Dynamic sites were most prominently enriched for overlap with enhancer and TFBS elements (p < 0.01) (Fig. 3C). Second, we determined whether the dynamic chromatin sites were evenly distributed across the genome. We observed significant clustering (p < 0.05) of dynamic sites suggesting a non-random distribution of these sites. Gene set enrichment analysis of the clustered sites identified processes relevant to foam cell formation and cellular lipid handling (Fig. 3D). Further, we performed motif analysis using the genomic sequence within dynamic chromatin sites. We identified several motifs enriched in these sequences, including JunD/AP1, NFE2L2 and EGR1 (S8 and S9 Tables). These results suggest dynamic chromatin sites are enriched for regulatory potential.

### Macrophage enhancers undergo major oxLDL-induced changes

**Fig 4:**
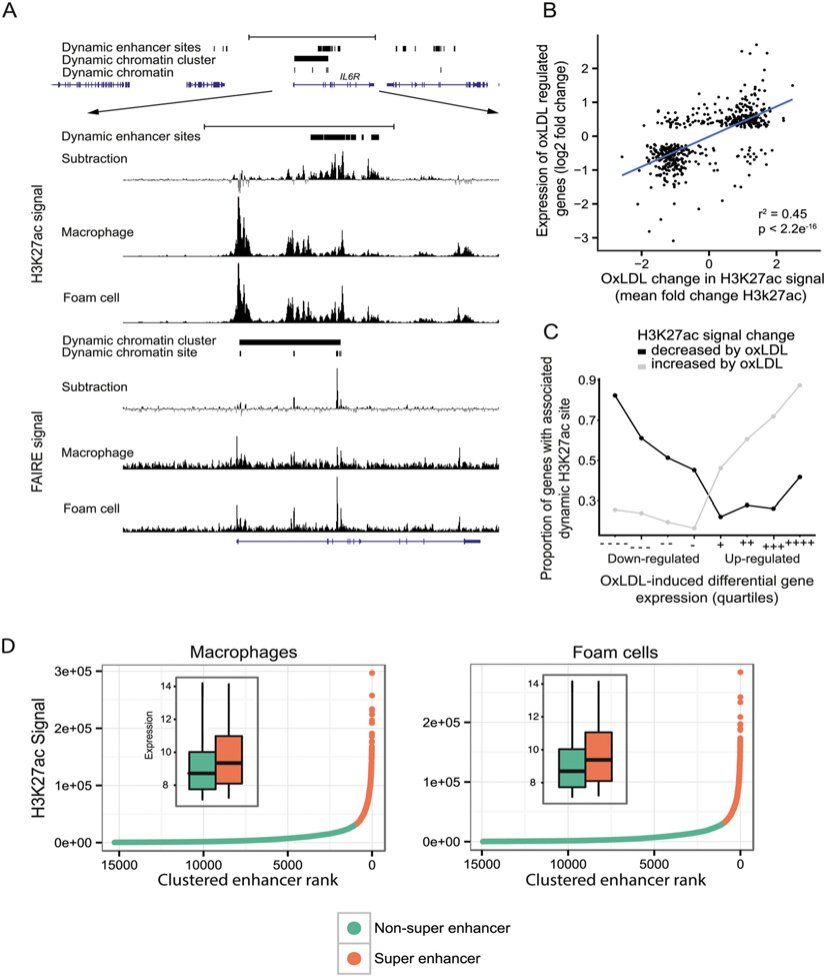
OxLDL-regulated dynamic enhancer signals correlate with expression changes in local genes. (**A**) Open chromatin (FAIRE) and enhancer (H3K27ac) profile at the *IL6R* locus showing a dynamic chromatin cluster that overlaps a large, intronic dynamic enhancer site with increased signal in oxLDL-treated cells. (**B**) Correlation between fold change in gene expression for differentially expressed genes and mean fold change in enhancer signal for dynamic sites annotated to their nearest gene (r^2^ = 0.45, Pearson r = 0.67). (C) The proportion of the genes in each quartile that are associated with a dynamic enhancer site is plotted. Differentially expressed genes were split into oxLDL up and down regulated genes and further split into quartiles based on fold change in expression (-/+ and—-/++++ indicate least and most differential quartiles, respectively). The proportion of differentially expressed genes in each quartile that are associated with a directionally concordant change in a dynamic enhancer site is correlated with the magnitude of gene expression change. (**D**) Enhancers in macrophages or foam cells were aggregated if within 12.5kb and plotted according to H3k27ac signal rank. The insets show gene expression (log2, normalized) for genes overlapping either enhancers within super enhancer domains or other enhancers.

As the majority of dynamic chromatin sites were distal to known promoters, we hypothesized that many of these sites represented enhancer elements involved in the macrophage response to oxLDL and thus in foam cell identity. We, therefore, mapped the enhancer landscape in macrophages and foam cells using ChIP-seq for the histone modification H3K27ac, which is known to mark active enhancers [24,30]. We observed significant oxLDL-induced changes in H3K27ac signal at 39,194 sites, termed ‘dynamic enhancer sites’, 95% of which were distal to known promoter regions (Fig. 4A, S10 Table). We found a strong positive correlation between the change in enhancer signal and the change in expression of the nearest differentially expressed gene (r^2^ = 0.45; p < 2.2x10^-16^) (Fig. 4B, 4C). We identified ‘super-enhancer’ elements, which are spatially clustered enhancers that, in other contexts, are relevant to the expression of cell-type specific genes [31]. We identified 1,081 and 1,098 super enhancers in macrophages and foam cells, respectively (Fig. 4D, S11 and S12 Tables). Genes overlapping or adjacent to super enhancers had significantly higher expression than those associated with other enhancers (Fig. 4D inset). Of the 1098 foam cell super-enhancer clusters, 213 were evident as super-enhancer clusters only in foam cells and genes nearest to or overlapping them had significantly higher expression in foam cells compared to macrophages (p = 4.61x10^-10^). The analysis of enhancers indicates that oxLDL-induced changes in gene expression are mediated in large part through changes in local enhancer activity.

**Fig 5:**
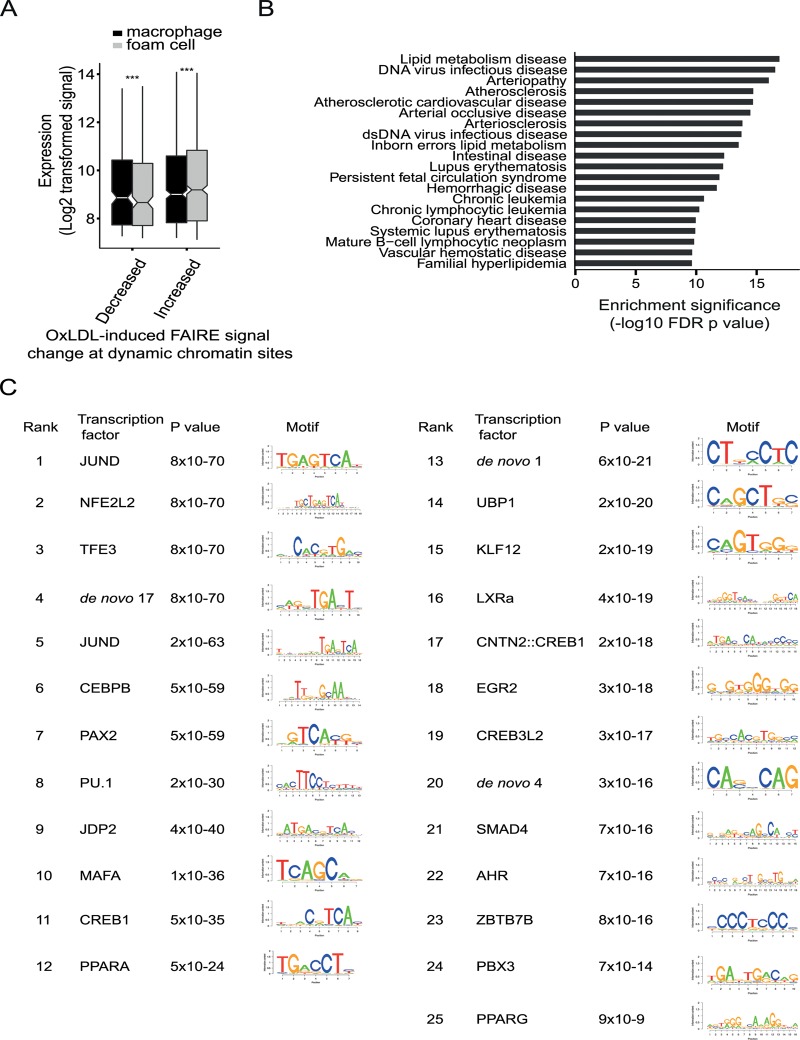
OxLDL induces changes in both open chromatin and enhancer status at a subset of sites with important regulatory function. (**A**) The oxLDL regulated change in open chromatin, at sites which also have changes in enhancer signal, is correlated with expression of the nearest gene (*** p < 0.0005). (**B**) The refined subset of sites with dynamic open chromatin and dynamic enhancer status shows enrichment for proximity to disease-associated gene sets linked with atherosclerosis. (**C**) The subset of sites with dynamic open chromatin and enhancer signal was analyzed for transcription factor motif enrichment and the top 25 motifs are shown with the p values for enrichment. Motifs were identified using SeqPos by comparison with known motifs from the Cistrome database and by identification of *de novo* motifs. SeqPos clusters similar motifs and the transcription factors listed are representative of each cluster. The position weight matrices for the *de novo* motifs are provided in S13 Table.

We next identified sites that had significant oxLDL-induced changes in both chromatin signal and enhancer signal. 1743 dynamic chromatin sites overlapped one or more of 2004 dynamic enhancer sites. Of these 2004 enhancer sites there was concordance in the direction of change in enhancer and open chromatin signals in 1817 sites (90.6%). These 1743 dynamic chromatin sites were, in turn, significantly associated with a directionally concordant change in expression of the nearest genes and chromatin signal (oxLDL-induced chromatin sites p = 6.9x10^-27^, oxLDL-suppressed chromatin sites p = 0.0004, Fig. 5A). We also observed enrichment among genes nearest to these 1,743 dynamic sites for gene sets related to atherosclerotic vascular disease (Fig. 5B). These results demonstrate that a discrete number of sites with changes in both chromatin accessibility and enhancer signal are associated with disease-relevant changes in gene expression.

### Dynamic enhancer sites identify C/EBP-beta as a novel oxLDL-regulated transcription factor in macrophages

We used the dynamic chromatin and enhancer sites to identify potential upstream transcriptional regulators driving oxLDL-induced changes in macrophage chromatin and gene expression. We performed motif enrichment analysis using the genomic sequence of the 1,743 sites with changes in both chromatin and enhancer signal (Fig. 5C, S13 Table). We observed enrichment for AP1-related factors, such as JunD, which bind enhancer elements, and NFE2L2, which is involved in the anti-oxidant response [17,21]. We also observed enrichment for factors regulated directly by cellular lipids, including LXRA and PPARG [32]. Notably, we observed enrichment for several predicted C/EBP motifs including C/EBP-beta, which is a known myeloid transcription factor, but has no known role in foam cell formation [33]. These results suggest hypotheses concerning the role of specific regulatory proteins in the macrophage response to oxLDL, including proteins such as C/EBP-beta with no prior links to this process.

**Fig 6:**
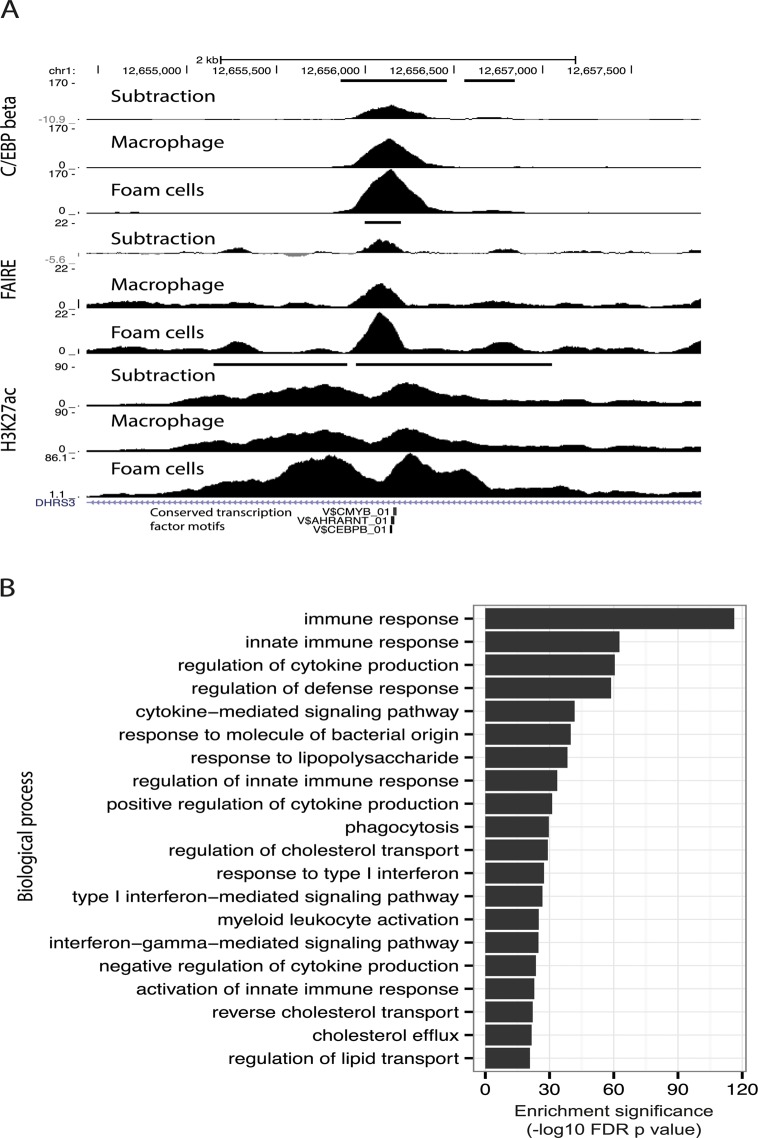
C/EBP-beta binding is responsive to oxLDL. (**A**) Track view of a dynamic open chromatin and enhancer site which also overlaps a dynamic C/EBP-beta binding site with increased C/EBP-beta binding in foam cells (horizontal black bars indicate dynamic sites). (**B**) Enrichment of gene sets associated with different biological processes for dynamic C/EBP-beta binding sites.

Given enrichment for C/EBP-beta motifs in dynamic enhancer sites, in addition to up-regulation of the *CEBPB* gene itself after oxLDL exposure (S1B Table, Fig. 5C), we hypothesized that C/EBP-beta regulatory activity in macrophages would be stimulated by oxLDL exposure. We, therefore, performed ChIP-seq for C/EBP-beta binding in macrophages before and after oxLDL exposure and consequent foam cell formation. We observed 10,212 genomic sites with significantly different C/EBP-beta binding signal between cell types, almost all of which (97%, 9,907 sites) had increased signal in foam cells (S14 and S15 Tables, Fig. 6A). Dynamic C/EBP-beta sites were enriched for proximity to genes relating to innate immune functions (Fig. 6B), suggesting that C/EBP-beta may be involved in inflammatory processes triggered by oxLDL during foam cell development.

### CAD-associated genetic variants are enriched in regulatory DNA sites altered by oxLDL

**Fig 7:**
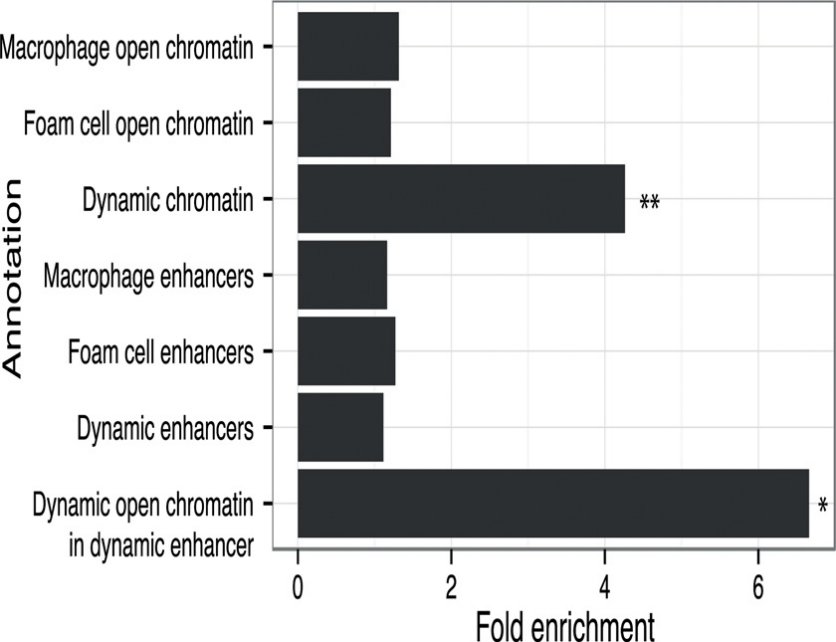
CAD loci are enriched in oxLDL-regulated open chromatin sites. Fold enrichment of CAD loci with variants overlapping different types of regulatory features compared to background loci (* p < 0.05, ** p < 0.005).

Disease-associated genetic variants may alter transcriptional regulation by affecting transcription factor binding to regulatory DNA elements [9]. Therefore, we sought to profile the relationship between the oxLDL-induced changes we had identified in macrophages and genetic variants associated with CAD risk. We identified 45 independent CAD-associated genomic loci and catalogued the reported index SNP and all SNPs in high LD (r^2^ > 0.8) [34]. We then intersected macrophage and foam cell epigenomic data with the resulting set of variants at CAD-associated loci (Table 2, S2 and S16 Tables). At 22 of the 45 CAD loci, one or more variants lay within an open chromatin or enhancer site in macrophages and/or foam cells. We tested for enrichment of variants at CAD-associated loci in dynamic sites compared to the expected overlap derived from a background set of matched non-CAD GWAS loci (see Methods). We observed significant enrichment of CAD-associated loci in dynamic chromatin sites (fold = 4.26; binomial p = 0.0027) (Fig. 7), an effect that was stronger when considering sites with both dynamic chromatin and enhancer signal (fold = 6.66; p = 0.036). Conversely, we observed no significant enrichment when considering chromatin sites in macrophages and foam cells alone (p = 0.19, p = 0.31). These results suggest that variants at CAD-associated loci are specifically enriched in the subset of macrophage regulatory sites altered by oxLDL exposure.

**Table 2:** Number of CAD loci and individual SNPs overlapping regulatory elements in macrophages and foam cells.

Feature type		Number of CAD loci	Number of SNPs
Open chromatin	Macrophage	12	26
	Foam cell	12	26
Enhancer	Macrophage	18	109
	Foam cell	19	114
Dynamic chromatin		6	9
Dynamic enhancer		9	38

CAD-associated variants at the *IL6R* and *PPAP2B* loci were within overlapping dynamic chromatin and enhancer sites. At both loci the CAD-associated variants (rs7549338 and rs7553796 at *IL6R;* rs72664324 at *PPAP2B*) overlap single dynamic sites that had increased signals after oxLDL exposure; further, both *IL6R* and *PPAP2B* had significantly up-regulated expression in response to oxLDL (S4 Fig). *IL6R* encodes the receptor for the pro-inflammatory cytokine interleukin-6 and circulating levels of a soluble form of IL6R have been associated with coronary artery disease [35,36]. *PPAP2B* encodes lipid phosphate phosphohydrolase 3 (LPP3), an enzyme that metabolizes and so deactivates pro-inflammatory mediators [37]. The mechanism through which the *PPAP2B* locus influences CAD risk is unknown and the expression pattern and regulation of *PPAP2B* by oxLDL has not been studied previously. These results demonstrate that dynamic sites can highlight candidate causal variants and genes at CAD-associated loci.

### CAD-associated variant rs72664324 demonstrates allelic differences in oxLDL-induced enhancer activity

**Fig 8:**
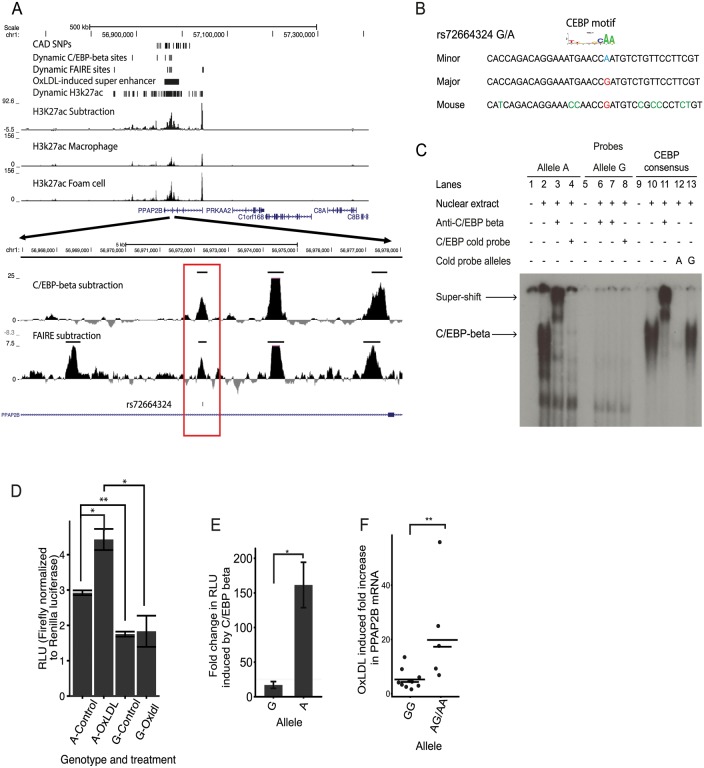
An intronic SNP at the *PPAP2B* locus regulates enhancer activity and oxLDL-induced expression of *PPAP2B*. (**A**) Chromatin profile at the *PPAP2B* locus where rs72664324 is in a dynamic chromatin, enhancer and C/EBP beta site (red box). The region is part of an oxLDL-induced super enhancer. (**B**) Comparison of the human rs72664324 alleles and the corresponding mouse sequence with the CEBP motif aligned above. (**C**) EMSA demonstrating that only the A allele at rs72664324 binds nuclear protein, which is shown to be C/EBP beta by a super-shift in the presence of anti-C/EBP beta antibody. Also, only a cold probe with the A allele competes off binding to a C/EBP consensus probe. (**D**) Luciferase reporter assays in primary human macrophages and foam cells with a reporter element containing rs72664324 with either allele (* p < 0.05, ** p < 0.005). (**E**) Effect of C/EBP beta overexpression on luciferase reporter activity with either the A or G allele relative to empty vector (* p < 0.05). (**F**) Induction of *PPAP2B* expression by oxLDL in primary human macrophages from individuals with allele G or at least one copy of the A allele (long lines indicate mean, short lines indicate median, Mann-Whitney U test, p = 0.0013, GG n = 10, GA n = 2, AA n = 6).

We sought to identify and characterize candidate causal CAD-associated variants at the *PPAP2B* locus. A single CAD-associated variant rs72664324 overlapped a dynamic chromatin and enhancer site, which in turn lies in an oxLDL-induced ‘super-enhancer’ cluster (Fig. 8A).

We first investigated whether the alleles at rs72664324 displayed differential predicted transcription factor binding. Sequence motif analysis identified predicted binding for both alleles of rs72664324 to several factors such as STAT5A, GR and NFY, as well as allele-specific binding of C/EBP-beta and -alpha to the A allele (Table 3, S17 Table). Electrophoretic mobility shift assays (EMSA) demonstrated nuclear protein binding to the protective A allele at rs72664324, but not to the risk G allele (Fig. 8B, 8C). Competition EMSAs with cold probes corresponding to consensus binding sequences for each factor showed that the C/EBP consensus sequence reduced nuclear protein binding to the A allele (S5 Fig and Fig. 8C). Further, antibodies to C/EBP-beta, but not to C/EBP-alpha, were able to super-shift the band seen with the A allele (S6 Fig and Fig. 8C). The super-shift seen with anti-C/EBP-beta was the same for the A allele sequence and the consensus C/EBP sequence, demonstrating that the A allele sequence and the consensus sequence both bind C/EBP beta with a similar affinity. These results indicate that compared to the G allele, the A allele of rs72664324 preferentially binds C/EBP-beta. We also observed binding of C/EBP-beta to the dynamic chromatin and enhancer region overlapping rs72664324 in chromatin (Fig. 8A). We observed greater C/EBP-beta signal in individuals homozygous for the A allele compared to the G allele (3.7 fold) and oxLDL increased the C/EBP-beta signal by an average of 2.6-fold at this site, consistent with the direction of the oxLDL-induced dynamic chromatin and enhancer site (Fig. 8A). These results demonstrate that the A allele of rs72664324 increases binding of C/EBP-beta at an oxLDL-dynamic enhancer.

**Table 3:** Differential transcription factor motif affinity at the rs72664324 site for the risk G allele and the protective A allele.

Rank	Difference log(p) for two sequences	A allele p value	G allele p value	Matrix ID (Transfac)	Transcription factor
1	1.71	0.0137	0.693	M00190	CEBP
2	1.48	0.0263	0.794	M00116	CEBPA
3	0.978	0.0452	0.429	M00621	CEBPDELTA
4	0.874	0.0102	0.0762	M01147	DMRT2
5	0.828	0.104	0.698	M01334	NKX11
6	0.822	0.0471	0.312	M00309	ACAAT
7	0.821	0.0498	0.33	M00775	NFY
8	0.816	0.0902	0.591	M00109	CEBPB
9	-0.814	0.936	0.144	M00975	RFX
10	-0.802	0.199	0.0314	M00792	SMAD

We next tested the effects of rs72664324 on oxLDL-induced enhancer activity using luciferase reporter assays in primary human macrophages and foam cells. We observed increased enhancer activity with the A allele compared to the G allele in both macrophages (1.73 fold, p = 0.0005) and foam cells (2.25 fold, p = 0.025) (Fig. 8D). Furthermore, we observed a greater oxLDL-induced increase in enhancer activity at the rs72664324 site with the A allele (1.43 fold, p = 0.03) compared to the G allele (1.098, p = 0.78) (Fig. 8D). Overexpression of C/EBP-beta increased transcriptional enhancer activity of the A allele significantly more than that of the G allele (p = 0.012) (Fig. 8E). These results suggest that, compared to the G allele, the A allele preferentially increases enhancer activity upon oxLDL induction through increased C/EBP-beta binding.

We obtained primary macrophages from healthy individuals with different rs72664324 genotypes. We observed significantly higher oxLDL-induced PPAP2B expression in macrophages from individuals with at least one copy of the A allele compared to those with only the G allele (p = 0.0013) (Fig. 8F). The A allele of rs72664324 is in LD with the protective allele of the reported index SNP at this locus, so higher PPAP2B expression is associated with reduced CAD risk.

### OxLDL up-regulates *PPAP2B* expression and influences pro-inflammatory mediators

**Fig 9:**
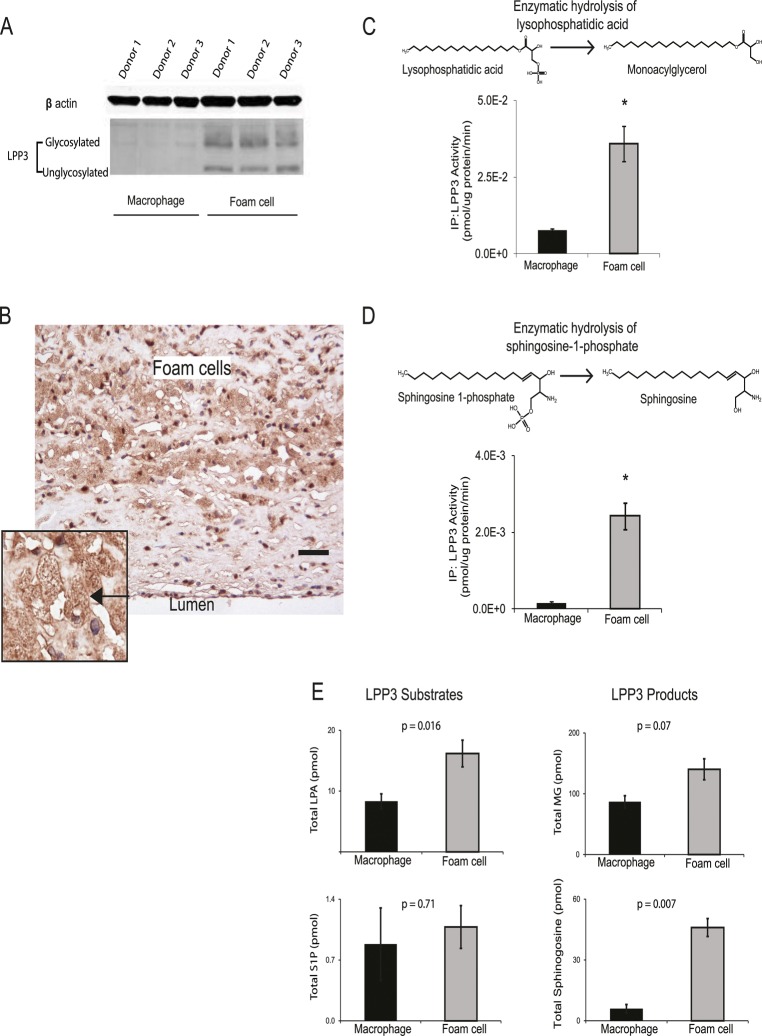
OxLDL exposure induces *PPAP2B*-encoded LPP3 expression and activity in foam cells. (**A**) Western blotting demonstrates up-regulation of glycosylated and non-glycosylated LPP3 protein (as indicated) in macrophage-derived foam cells (data from 3 donors shown). (**B**) Human atherosclerotic plaque contains an abundance of LPP3-expressing foam cells (brown stain, nuclei counterstained blue with haematoxylin, scale bar indicates 50 um, inset shows close-up of foam cell—arrowed). All 5 cases immunostained showed similar LPP3 expression in foam cells. (**C and D**) OxLDL exposure induced increased specific activity of LPP3, measured by the degradation of receptor active species lysophosphatidic acid (LPA) to mono-acylglycerol (MG) (**C**) and sphingosine-1-phosphate (S1P) to sphingosine (**D**) (n = 3 donors, * p < 0.05). (**E**) OxLDL induced changes in the levels of LPP3 substrates and products (n = 3 donors).

**Fig 10:**
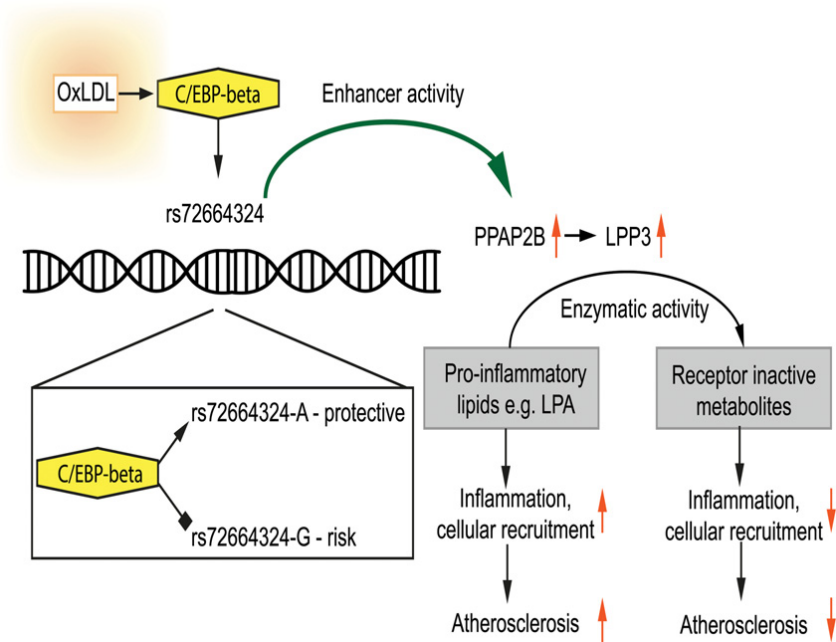
Model showing the interplay between oxLDL, C/EBP beta and rs72664324 on expression of *PPAP2B*. OxLDL-induced C/EBP beta binding is greater to the A allele and leads to increased induction of *PPAP2B* in response to oxLDL compared to the G allele. *PPAP2B* encodes LPP3 whose enzymatic activity reduces pro-inflammatory signalling mediated by LPA.

The role of *PPAP2B* in the response of primary human macrophages to oxLDL has not been studied previously. Given the influence of the CAD risk variant rs72664324 on PPAP2B expression in the macrophage response to oxLDL and our observation that *PPAP2B* was the seventh most up-regulated gene in response to oxLDL exposure (Table 1), we studied the effects of oxLDL on the activity of the resulting protein product LPP3, and the expression pattern of LPP3 in atherosclerotic lesions. LPP3 is an enzyme that dephosphorylates and thus deactivates pro-inflammatory mediators including lysophosphatidic acid and sphingosine 1-phosphate [37–39]. Protein expression of LPP3 in primary human macrophages was strongly increased by oxLDL treatment, as demonstrated using western blotting and mass spectrometry (Fig. 9A). Immunohistochemistry for LPP3 in human arterial atherosclerotic plaques confirmed that foam cells express LPP3 *in vivo* and that these cells constitute the major source of LPP3 within the plaque (Fig. 9B). Enzymatic activity of LPP3 against both LPA and S1P was strongly induced in primary macrophages by oxLDL exposure (Fig. 9C, 9D). Mass spectrometry-based analysis also identified significant oxLDL-induced changes in substrates and products of LPP3 (Fig. 9E). These results indicate that the CAD protective A allele of rs72664324 at the *PPAP2B* locus increases the transcriptional enhancer response to oxLDL in macrophages, resulting in altered LPP3 activity, which in turn promotes increased metabolism of pro-inflammatory mediators within atherosclerosis lesions (Fig. 10).

## Discussion

Both environmental and genetic factors influence an individual’s risk of developing coronary artery disease. We have used epigenetic techniques for mapping chromatin structure at cis-regulatory elements to study the interacting effects of an environmental stimulus and risk-associated genetic variants.

Overwhelming evidence indicates that LDL is the major environmentally influenced contributor to the development of atherosclerosis. In humans circulating levels of LDL are influenced by diet and are tightly correlated with CAD risk; lowering LDL lowers risk [4–6]. In various animal models, diets that elevate LDL accelerate atherosclerosis [40]. The most striking cellular effect of lipid in atherosclerosis results from the interaction of oxLDL with macrophages. OxLDL is formed by modification of protein and lipids within LDL particles that have become trapped in the arterial vessel wall [2,41]. Macrophages within the arterial wall take up this oxLDL in an uncontrolled manner, predominantly via scavenger receptors [2,42]. Progressive oxLDL uptake results in the formation of lipid-laden foam cells [2,42]. Foam cells contribute to the inflammatory nature of the atherosclerotic lesions in various ways, as they have reduced motility, release inflammatory cytokines, chemokines, degradative enzymes, reactive oxygen species and ultimately undergo apoptosis with release of a cocktail of further pro-inflammatory cellular contents [2,15,42].

In this study we have mapped the effects of oxLDL on chromatin remodeling and gene expression in macrophages and integrated these data with the results of GWAS in CAD to identify candidate functional variants. Further, these results provide a detailed map of the epigenetic changes arising from exposure of macrophages to oxLDL and so provide a rational basis for the study of approaches to ameliorate foam cell formation.

Open chromatin mapping with FAIRE-seq identifies the locations of all types of cis-regulatory elements and can be used to study changes in chromatin structure in cells subjected to a variety of stimuli or undergoing ontogenetic change [23,43–46]. However, as FAIRE-seq does not directly provide information about the function of DNA elements, we simultaneously undertook ChIP-seq for H3K27ac which marks active enhancers [24,47]. Identifying the subset of open chromatin sites with active enhancer status is important because they play a central role in gene regulation, particularly the control of cell-type specific gene expression [48]. H3K27ac also marks promoter sites, which are easily distinguishable by their genomic context.

FAIRE-seq derived open chromatin sites that vary between macrophages and foam cells contain regulatory DNA sequences that play a role in oxLDL-induced foam cell formation. We found that the number of open chromatin sites that were modified by oxLDL was substantially greater than the number of differentially expressed genes. This is likely to reflect the combinatorial effects of sets of these dynamic chromatin sites and clusters of spatially linked sites were enriched around genes whose expression was altered by oxLDL. Although we detected a significant change in open chromatin structure at oxLDL-regulated promoters, the fold change in signal was smaller than that seen at non-promoter sites. This is consistent with data indicating that promoter chromatin structure is largely set at an early stage during cell ontogeny as 70% of promoter sites remained constant during adipogenesis compared to only 25–40% of non-promoter sites [46].

OxLDL also induced widespread changes in the H3K27ac enhancer signal and a close correlation was demonstrated between differential gene expression and the mean change in enhancer signal across multiple dynamic H3K27ac sites annotated to their nearest gene. Other studies have found that not all sites of H3K27ac signal are associated with an open chromatin site [49]. We found that at a subset of 1743 non-promoter sites there were oxLDL-induced changes in both the open chromatin site and the surrounding enhancer marks and that the changes were directionally concordant at over 90% of these sites. The value of combining H3K27ac and FAIRE-seq data in this way is evident from the increased strength of the relationship of these dually identified sites with nearby gene expression [50]. Similarly, we found a robust relationship between the oxLDL-induced change in FAIRE signal at these sites and expression of the nearest gene. The importance of this set of sites was underlined by their proximity to gene-sets strongly enriched for genes known to be involved in coronary artery disease. As a subset of enhancers for further study, these sites represent a valuable prioritized set, since the dynamic open chromatin sites pinpoint the precise locations where altered transcription factor binding must occur.

Given the strong association of these dynamic enhancers with gene expression, identifying the transcription factors that bind them would very valuable in defining the upstream regulatory pathways influenced by oxLDL. Within the 1,743 sites we identified a distinct signature of transcription factor binding using motif analysis. As validation of our approach, we found enrichment for binding sites for NRF2 (NFE2L2) and PPARG, which are both known to be associated with foam cell formation. NRF2 mediates the oxidative stress response to oxLDL and PPARG is a nuclear receptor for constituents of oxLDL [51,52]. The list of enriched transcription factors included C/EBP-beta and AP1, which are both known to act as pioneer factors and so can bind relatively closed chromatin, causing chromatin remodeling and thus allowing further transcription factors to bind [21]. Using ChIP-seq we confirmed oxLDL increased C/EBP-beta binding at multiple genomic sites. In keeping with these results of an effect of oxLDL on C/EBP-beta, the saturated fatty acid palmitate has been shown to induce inflammatory changes in murine macrophages by a C/EBP-beta-dependent mechanism [53]. In adipocytes C/EBP beta causes chromatin remodeling which opens up sites for PPARG binding [49]; our data raise the possibility of a similar process in foam cell formation with oxLDL exposure resulting in C/EBP-beta opening up chromatin for other transcription factors [49].

Profiling the co-localization of regulatory sites with genetic risk variants identified by GWAS has been useful in the identification of cell types in which disease risk variants operate [28,54]. Given the established role of macrophages and foam cells in atherosclerosis it was initially surprising that we found no enrichment for CAD risk loci in either context alone. However, we found that those sites that underwent oxLDL-induced changes in chromatin accessibility were enriched for CAD-associated loci. This demonstrates that the response to oxLDL, with concomitant foam cell formation, induces chromatin changes at the sites of a subset of CAD variants and provides a cellular context for risk variants at these sites to operate in altering disease risk. Our data show that the regulatory capacity of key SNPs in dynamic sites is actuated in macrophages by an environmentally influenced stimulus, oxLDL, so demonstrating the mechanism for a direct interplay between environmental and genetic risk factors. To our knowledge this is the first time enrichment for CAD loci has been demonstrated in open chromatin in an adult cell type.

The *PPAP2B* CAD locus was of particular interest because oxLDL induced more open chromatin, greater enhancer activity and up-regulated PPAP2B expression. Our finding that PPAP2B and it protein product, LPP3 were up-regulated by oxLDL fits with a growing body of evidence implicating dysregulation of its substrates, LPA and S1P in atherosclerosis. LPP3 hydrolyzes LPA and S1P to their non-receptor active forms [37,55]. LPP3 is known to have a role in vascular development and endothelial integrity, but its role in macrophages and foam cells remains unknown [56,57]. We found that *PPAP2B* was one of the most up-regulated genes in foam cells, was abundant in human plaque foam cells and its specific enzymatic activity towards LPA and S1P in macrophages was increased by oxLDL.

LPA is an obligate intermediary of triglyceride and glycerophospholipid synthesis and an extracellular ligand for 6 different LPA receptors [58,59]. There is an accumulation of LPA in plaques in both human atherosclerotic disease and murine models of atherosclerosis [60–63]. LPA has pro-atherogenic effects on most of the cells involved in atherosclerosis, promoting vascular smooth muscle proliferation, endothelial cell adhesion molecule expression, and stimulating oxLDL uptake by macrophages [64]. In addition, LPA is a highly thrombogenic mediator and its release upon atherosclerotic plaque rupture can contribute to thrombotic occlusion of the artery [61]. Strategies that modulate LPA levels in plaques could, among other beneficial effects, reduce thrombogenicity.

S1P is a receptor active sphingolipid which signals via 5 G-protein linked cell surface receptors resulting in diverse effects including immune cell trafficking and angiogenesis [65]. Recent studies have sought to characterize the precise role of S1P in atherosclerosis using murine models in which S1P receptors have been knocked out. Mice deficient for S1P2R or S1P3R and ApoE have reduced numbers of macrophages and foam cells in plaques [66]. Bone marrow chimeras indicate that this effect in S1P2R deficient mice is dependent on hemopoeitic cells and S1P2R deficiency reduced macrophage inflammatory response. Nevertheless, the precise role of S1P in different aspects of atherosclerosis remains to be determined

Our immunohistochemical studies of human atherosclerotic plaque indicated that the predominant source of LPP3 was in foam cells. In keeping with an accumulation of LPA in atherosclerotic plaque, we found that foam cells contained significantly more LPA than macrophages. Taken together our findings suggest that by degrading LPA, LPP3 could have a role as a negative regulator of pro-inflammatory LPA signaling during the macrophage response to oxLDL and in foam cells.

The *PPAP2B* locus is well validated as a CAD risk locus and our data demonstrate that the risk allele reduced the induction of PPAP2B by oxLDL in macrophages [8,67,68]. This suggests a plausible model whereby the risk allele lowers transcription of PPAP2B in response to oxLDL exposure in macrophages (Fig. 10). This in turn would lower levels of LPP3 in foam cells, resulting in higher levels of LPA in plaque and consequently increased pro-inflammatory signaling, vascular smooth muscle proliferation, retention of foam cells in lesions and thrombogenicity. Our data demonstrate that an effect on PPAP2B transcription may in part be mediated through rs72664324, a SNP that is in high linkage disequilibrium with the reported GWAS SNP (rs17114036). The risk allele at rs72664324 reduces C/EBP-beta binding at this site and so reduces the enhancer activity of the site. The overall mechanism we propose is in keeping with that shown for two other CAD loci where rs12740374 affected SORT1 transcription and SNPs at the 9p21 locus altered STAT1 binding and interferon gamma signaling [9,10].

Further evidence suggests that rs72664324 is a causal SNP. None of the other 20 SNPs in high linkage disequilibrium were in the coding sequence or even the 3’UTR, where altered miRNA binding is an additional mechanism by which CAD SNPs might act [11]. The chromatin structure at the reported SNP, rs17114036, remained closed before and after oxLDL exposure. Moreover, no other SNP in high linkage disequilibrium at the locus was within an open chromatin site before or after oxLDL treatment. Interestingly, we found that the DNA sequence at the dynamic chromatin site containing rs72664324 has been highly conserved across vertebrate species. Active enhancers have been associated with the production of small RNA transcripts and using CAGE-seq to measure enhancer transcripts across the genome in hundreds of predominantly ‘healthy’ primary cells and tissues, the only enhancer RNA that overlapped any of the 21 SNPs in high linkage disequilibrium with the reported risk SNP was that from the rs72664324 site [69]. This enhancer was only detected in monocytes, the precursors of macrophages [69]. Finally, we show that the presence of the protective A allele at the rs72664324 site enhances the upregulation of PPAP2B expression that is triggered by oxLDL in primary macrophages. Nevertheless we cannot exclude that other linked SNPs may be important, especially since our primary open chromatin data and enhancer mapping was performed in individuals homozygous for the major allele of the CAD reported SNP.

Our data indicate that a CAD risk variant operates to alter the response of macrophages to oxLDL exposure by altering binding of C/EBP beta to an enhancer site regulating PPAP2B expression. This will influence inflammatory and other aspects of the atherosclerotic disease process. Targeting the activity of PPAP2B-encoded LPP3 in macrophages and foam cells is a plausible therapeutic strategy. Future studies could directly address the role of *PPAP2B* in the response to oxLDL using murine models in which *PPAP2B* is selectively knocked out from myeloid cells. Overall, our study establishes a link between CAD genetic susceptibility, the macrophage response to atherogenic lipid and receptor active lipid signaling. This study demonstrates the utility of chromatin and enhancer mapping in primary human cells before and after a pathogenic environmental stimulus and this approach may have applications in the study of other diseases.

## Materials and Methods

### Cell culture

Ethical approval for the study was obtained from the NHS Research Ethics Committee (South Central-Hampshire B, reference 13/SC/0392) and all participants provided informed consent.

CD14^+^ monocytes were isolated from healthy human volunteers by centrifugation of peripheral blood over Ficoll-Paque PLUS (GE Healthcare LifeSciences, Piscataway, NJ) followed by extraction with magnetic beads conjugated to anti-CD14 antibody (Miltenyi Biotec, Bergisch Gladbach, Germany). Monocyte purity was assessed by flow cytometry using anti-CD14 antibody (AbD Serotec, Raleigh, NC) and was ≥ 95%. Cells were maintained in RPMI 1640 medium with 10% fetal calf serum, 4 mM L-glutamine, 50 units/ml penicillin and 50 μg /ml streptomycin (Sigma, St Louis, MO), supplemented with 50 ng/ml macrophage colony stimulating factor (eBioscience, San Diego, CA). After 7 days these macrophages were treated with either control buffer (1 mM ethylenediaminetetraacetic acid (EDTA), 25 μm CuCl_2_, phosphate-buffered saline (PBS)) or 50 μg/ml oxLDL for 48hrs. Oil red O staining of intracellular lipids and measurement of cellular cholesterol and cholesterol ester content by mass spectrometry confirmed foam cell formation in oxLDL-treated cells. Viability of cells was confirmed to be > 98% using the Invitrogen LIVE/DEAD fluorescent microscopy kit (Life Technologies, Carlsbad, CA). THP1 cells (ATCC) were maintained in RPMI 1640 with 10% fetal calf serum, 4 mM L-glutamine, 50 units/ml penicillin and 50 μg/ml streptomycin. THP1 cells were treated with 50 ng/ml phorbol myristate acetate (PMA, Sigma) for 48 hours to obtain adherent macrophage cells.

### Preparation of oxLDL

LDL (d 1.019–1.063 g/ml) was freshly isolated from human plasma by ultracentrifugation using a discontinuous potassium bromide gradient [70]. Precautions were taken to prevent endotoxin contamination and maintain sterility. LDL was extensively dialyzed against PBS in sterile gamma-irradiated cartridges (Pierce, Rockford, Il) and protein concentration was measured with the BCA method (Pierce, Rockford, Il). LDL was oxidized by incubation with 25 μm CuCl_2_ at 37° C for 18 hours. Oxidation was confirmed by thiobarbituric reactive substances assay (Caymen Chemical, Ann Arbor, MI). Oxidation was terminated by addition of 1 mM EDTA and storage at 4#x00B0;C. OxLDL was used within 2 weeks of production. For endotoxin testing, samples were first heated to 75°C for 15 minutes to remove the plasma inhibitor and then assayed using the gel clot method according to the manufacturer’s instructions (Associates of Cape Cod, East Falmouth, MA). Levels were < 0.1 EU/ml.

### FAIRE

FAIRE was performed as described with modifications [22]. Five million primary human macrophages or foam cells were cross-linked, lyzed and sonicated (30 pulses of 15 seconds at maximum intensity using a BioRuptor (Diagenode, Denville, NJ)). Nucleosome-depleted DNA was extracted using 4 phenol-chloroform extractions and purified by ethanol precipitation. Three independent biological replicates were produced using cells from three healthy donors. Donors were homozygous for the reference allele of rs17114036/rs72664324.

### ChIP

ChIP was performed using the Invitrogen Magnify kit (Invitrogen) according to the manufacturer’s instructions using 200,000 primary human macrophages or foam cells. Cells were cross-linked with formaldehyde for 10 minutes on ice, lyzed and sonicated (32 pulses of 15 seconds, maximum intensity, Diagenode Bioruptor). H3K27ac-enriched DNA was immunoprecipitated using rabbit polyclonal anti-histone H3 (acetyl K27) antibody (ab4729, Abcam, Cambridge, MA). ChIP was performed in technical duplicates (pooled—total 400,000 cells) and two independent biological replicates were produced from two donors. Donors were homozygous for the reference allele of rs17114036/rs72664324. C/EBP-beta enriched DNA was immunoprecipitated using rabbit polyclonal anti-C/EBP-beta antibody (SC-150X, Santa Cruz, Santa Cruz, CA). ChIP was performed in technical duplicates (pooled total 400,000) cells and 4 biological replicates were produced from 4 donors (2 homozygotes for the reference allele and 2 for the non-reference allele of rs17114036/rs72664324).

### Library preparation and sequencing

Libraries were generated from gel-purified ~200 bp DNA fragments. After adapter ligation and PCR-based amplification, samples were sequenced on the Illumina HiSeq 2000 or 2500 platform (Illumina, San Diego, CA). 50 bp paired-end reads were mapped against the UCSC hg19 reference genome using STAMPY v1.021 [71]. For FAIRE and H3K27ac ChIP samples, reads were filtered (MAPQ minimum 15) in SAMTOOLS yielding at least 54 and 49 million reads respectively per sample [72]. For C/EBP-beta ChIP, paired end reads were mapped against the hs37d5 genome and filtered for a minimum MAPQ score of 4 and further filtered to remove duplicates (using the markduplicates tool in PICARD). For all subsequent analysis one read of each proper pair was retained along with all unpaired reads passing the requisite MAPQ threshold. Reads mapping to chrM, random contigs, unplaced contigs and ENCODE blacklisted regions were removed from subsequent analysis. To confirm reproducibility of FAIRE data, peaks were called on individual FAIRE samples using Fseq V1.84 (default parameters) and PeakDeck [73,74]. FAIRE peaks between replicates were intersected and shown to exceed ENCODE FAIRE-seq guideline standards (ENCODE and modENCODE Guidelines for Experiments Generating CHIP, DNase, FAIRE, and DNA Methylation Genome Wide Location Data Version 2.0[18]) before pooling for further analysis. ChIP samples peaks were called using MACS1.4.2 for H3k27ac and MACS2 for C/EBP-beta (versus input control DNA) and confirmed to exceed ENCODE guideline standards[75].

### FAIRE and ChIP-seq analysis

For FAIRE, dynamic chromatin sites between macrophages and foam cells were identified from pooled biological replicates using Diffreps V1.55 (windows 200 bp, step 20 bp, G-test, FDR < 2.5%) [76]. Clusters of dynamic chromatin were identified using the Diffreps HotSpot algorithm (p < 0.05). FAIRE-seq peaks were also determined for macrophages and foam cells individually using Fseq on pooled replicates (default settings, with threshold 8.5 for macrophages and 8 for foam cells). Peaks were merged if within 140 bp and filtered out if < 50 bp or > 5 kb in width. For ChIP dynamic H3K27ac enriched sites were identified with Diffreps using separate biological replicates (windows 500 bp, step 50 bp, FDR < 2.5%). ChIP-seq peaks were determined for each cell type individually using MACS1.4.2 on pooled biological replicates versus input control DNA with default parameters. Super enhancer sites were determined using the ROSE algorithm according to the originally established method[31]. For C/EBP beta ChIP-seq dynamic sites were identified from pooled biological replicates using Diffreps V1.55 (windows 200 bp, step 20 bp, G-test, FDR <2.5%, filtered on minimum 50 reads in dynamic site). Quantile normalized read counts in dynamic C/EBP-beta binding sites were determined for each genotype in order to compare signal at the rs72664324 dynamic site.

### Annotation of FAIRE and CHIP-seq sites

Genomic features were annotated using HOMER (V4.1), Diffreps and CEAS [76–78]. For average signal profiling at transcription start sites using CEAS, wig files of pooled biological replicates were produced using the Java genomics toolkit (https://github.com/timpalpant/java-genomics-toolkit) and subsets of ~200 genes were interrogated (for differentially expressed genes the top two quartiles of upregulated/downregulated genes based on fold change were used). To compare the FAIRE-seq signal between macrophages and foam cell promoters in different subsets of genes, the FAIRE-seq signal at all promoters was first quantified using HOMER (+ /- 1 kb transcriptional start site) and then quantile normalized. Signal at the promoters of all differentially expressed genes was then compared (paired Student’s t-test, significance p < 0.05) and displayed as boxplots. For correlating dynamic chromatin/dynamic enhancer sites with gene expression, the dynamic sites were annotated with the nearest gene and expression compared using a paired Student’s t-test, significance p < 0.05 and simple linear regression. For assigning dynamic enhancer status to dynamic chromatin sites, the dynamic chromatin sites were expanded by 300 bp in each direction to identify colocalization with histone marks of enhancer status that are on adjacent nucleosomes; finally the two datasets were intersected using bedtools. For genes with more than one Illumina microarray probe the differential expression data were extracted from the differential probe set with the most significant fold change between the two conditions. Gene set enrichment analysis of various dynamic chromatin/enhancer sites was performed using GREAT V2.0.2 and default parameters [79].

### Display in genome browser

For display of FAIRE-seq, H3K27ac or C/EBP-beta signal in the UCSC genome browser wig files were generated using ngs.BaseAlignCounts function in the Java genomics toolkit. Wig files were then normalized using the wigmath.scale function (default parameters) and macrophage signal was subtracted from foam cell signal to generate a track of dynamic signal.

### Transcription factor analysis

Transcription factor motif enrichment analysis was performed using SeqPos in the Cistrome Galaxy environment with the curated Cistrome motif database and *de novo* motif generator [80]. *De novo* motif position weight matrices are given in Tables S9 and S13.

### SNP enrichment analysis

We identified SNPs with genome-wide association (p < 5x10^-8^) to any trait in European individuals present in the GWAS catalogue. Index SNPs were pruned (r^2^ > 0.1 in CEU samples) so that each ‘locus’ was only represented by one index SNP to avoid counting redundant loci. Each index SNP was then used to identify variants in 1000 Genomes Project (1KG) pilot 1 data in high LD (r^2^ > 0.8) in CEU samples using HaploReg [81]. Thus, an associated ‘locus’ consists of an index SNP and the set of 1KG SNPs in high CEU LD. We then created a background set containing all qualifying loci, binned based on the number of total variants (index + high LD SNPs) in the locus.

We then identified published reports of CAD-associated variants with p < 5x10^-8^ in European samples. Where studies had reported different variants at the same locus we used the variant with the most significant p value [8,67,82–86]. For each CAD-associated variant we created loci containing 1KG variants in high LD (r^2^ > 0.8) in CEU samples [81].

Using the set of CAD-associated loci, we calculated the number of loci containing a variant overlapping a given annotation. We performed 100,000 permutations of the set of loci drawing from matching bins in the background set and recalculated the number of loci with a variant overlapping an annotation.

We then compared the observed number of loci, the total number of loci and the expected number obtained via permutation using a binomial test.

### Non-coding element enrichment

We obtained non-coding element data for ChromHMM chromatin state, TFBS, and multi-species conservation from the UCSC genome browser. For chromatin state data, we pooled ‘Enhancers’, ‘Promoters’ and ‘Insulators’ identified as such in any cell type.

We calculated the overlap of dynamic chromatin sites with each regulatory class, and then performed peak-shifting of the chromatin sites a random distance within a 10 kb window. We then re-calculated the overlap with the shifted sites, and derived a background distribution of expected overlap over 100 permutations. Fold-enrichment values were calculated relative to the background mean and p values for each overlap were obtained directly via permutation.

### Gene expression

Primary human macrophages and foam cells were lyzed and total RNA extracted using the TRIZOL RNA PLUS extraction kit (Life Technologies) with on-column Purelink DNAse treatment (Life Technologies). The integrity of the total RNA was analyzed on an Agilent Bioanalyzer 2100 (Agilent Technologies, Santa Clara, CA). Total RNA was reverse transcribed, amplified and biotinylated using the Illumina TotalPrep-96 RNA Amplification Kit (Ambion, Austin, TX). Biotinylated cRNA was hybridized to a single Human HT-12 V4 BeadCHIP (Illumina) at 58°C for 18 hours. The BeadCHIP was scanned using the Illumina Iscanner and data pre-processed using the Illumina Bead Studio to correct for local background effects, remove outlier beads, to compute average bead signal and SD for each probe and gene and to calculate p values. Hierarchical clustering showed that macrophages samples and foam cells samples clustered together respectively. The LUMI pipeline was used for data analysis. Data were transformed (variance-stabilizing transformation or log2 where indicated) and normalized (robust spline normalization). Differentially expressed genes were determined using both a paired macrophage-foam cell design and unpaired design (probe-centric) after removal of probes with absent expression. For differential expression an FDR-corrected p value threshold of 0.05 was deemed significant [87]. Enrichment p values for differentially expressed genes for candidate CAD genes were calculated using the binomial distribution. Heatmaps of differentially expressed genes were generated using the heatmap.2 function in R based on the subset of the genes with adjusted p value < 0.0001, and for genes with more than one microarray probe, the most significant probe was used (using the unpaired design). Gene set enrichment analysis was performed using GREAT.

For real time quantitative PCR (RTqPCR) 1 μg of DNaseI-treated total RNA was reverse transcribed using Bioscript (Bioline, London, UK) with random hexamers. RTqPCR was performed with SYBR Green reagents using a Step One Plus machine (Applied Biosystems, Foster City, CA) using technical triplicates and biological triplicates. Primers used: PPAP2B CO5547 TTCTGGCAGGATTTGCTCAA, CO5548 AGGGAGAGCGTCGTCTTAGTCTT; IL6R CO4830 GCATTGCCATTGTTCTGAGGTT, CO4831 ACCAGCTGCCCCAAAGAGT, GAPDH CO3744 TTGCCATCAATGACCCCTTCA, CO3745 CGCCCCACTTGATTTTGGA. For PPAP2B expression in genotyped healthy individuals; rs72664324-G n = 10, rs72664324-G/A n = 2, rs72664324-A n = 6.

### Genotyping

Genomic DNA was extracted from blood using Quick-gDNA minipreps (Zymo Research, Irvine, CA). Genotyping of the rs72664324 SNP was performed using a custom Taqman assay (Applied Biosystems, Foster City, CA). Forward primer AGGTGACCAGATATGCAAGTTGTC, reverse primer ACAGGGACTAGGACGAAGGAA. Allele specific MGB probes: A- AGGAAATGAACCAATGTCT, G- AGGAAATGAACCGATGTCT. Genotyping of rs17114036 was performed using an inventoried Taqman assay (C__33268873_10, Applied Biosystems). For allelic expression analysis, genotyped individuals were recruited from a cohort of healthy participants in the Oxford Biobank. Homozygous individuals at rs72664324 and rs17114046 all had alleles consistent with the known high LD such that rs72664324-AA was always associated with rs17114036-GG.

### Electrophoretic mobility shift assays (EMSA)

EMSA was performed using nuclear extracts from primary human macrophages treated with ^32^P gamma-ATP end-labeled double-stranded DNA probes (PerkinElmer, Waltham, MA) as previously described[88]. The forward strand probe sequences were: rs72664324-A aggaaatgaaccAatgtctgttcct, rs72664324-G aggaaatgaaccGatgtctgttcct, CEBP consensus TGCAGATTGCGCAATCTGCA, NFYA consensus CGTCTCCACCAATGGGAGGGCTGGGC, STAT5A consensus AGATTTCTAGGAATTCAATCC, GR consensus AGAGGATCTGTACAGGATGTTCTAGAT. For standard EMSA, 5 μg of nuclear extract was incubated with 100 fmol labeled probe in a 10 ul binding reaction containing 1 μg poly(dI-dC). For competition assays unlabeled probe at 100-fold excess was added to the binding reaction before addition of labeled probe. For super-shift assays the nuclear extract was pre-incubated with 1 μg antibody for 30 minutes on ice before probe was added. The following antibodies were used: rabbit polyclonal anti-C/EBP beta (SC-150, Santa Cruz), rabbit polyclonal anti-C/EBP alpha (SC-61, Santa Cruz).

### Luciferase reporter assays

To test enhancer activity at the rs72664324 locus in response to oxLDL, primary human macrophages were transfected with PGL4.23 reporter plasmids using nucleofection in biological triplicates as per the manufacturer’s protocol (Amaxa, Lonza, Portsmouth, NH). After 24 hours cells were then treated with oxLDL 50 μg/ml or buffer for a further 24 hours. To test enhancer activity in response to C/EBP beta over-expression, THP1 cells were cultured in 6 well dishes and transfected in at least triplicates using Lipofectamine LTX PLUS (Life Technologies) with 2.5 μg of vector for 36 hours. To control for transfection efficiency in each replicate, firefly luciferase plasmids were co-transfected with pRL-SV40 encoding Renilla luciferase (1/20 DNA amount compared to Firefly). Cells were lyzed for luciferase assay using the Dual Luciferase Reporter Assay System (Promega, Fitchburg, WI). The luciferase activity of each sample was normalized to Renilla luciferase activity and shown as relative light units (RLU). For each construct two individual clones were tested independently and representative data are shown. Data were analyzed using Student’s t-test on at least triplicates. rs72664324 reporter plasmids were generated in pGL4.23 for each allele, by cloning in four tandem repeats of GGAAATGAACCAATGTCT or GGAAATGAACCGATGTCT (Plasmid IDs; allele A = pOC1250, allele G = pOC1251). Human C/EBP beta LAP transcript was cloned into pCDNA3.1 (Plasmid ID = pOC1252). As control empty pCDNA3.1 was co-transfected with reporter plasmids. For co-transfections 1.25 μg of firefly plasmid and 1.25 μg of C/EBP beta LAP plasmid or control plasmid were transfected. All plasmids were verified by Sanger sequencing using BigDye (Life Technologies).

### Western blotting

LPP3 was detected using an extensively characterized and validated rabbit polyclonal anti-human LPP3 antibody and fluorescently multiplexed with beta-actin staining. Images shown are monochrome images of multiplex western blots [37,89–91].

### Measurement of LPP3 phosphatase activity

LPP3 was immunopreciptated from Triton X-100 extracted macrophage/foam cell proteins and phosphatase activity determined using heptadecanoyl lysophosphatidic acid and S1P as substrate and measuring heptadecanoyl monoacylglycerol and sphingosine produced using HPLC electrospray ionization tandem mass spectrometry. The reagents and methods employed have been described in detail elsewhere [37,39].

### Measurement of LPP3 substrates and products

Cholesterol, cholesterol ester and a series of LPP3 substrates and their cognate dephosphorylation products were quantitated in macrophage and foam cell lipid extracts by electrospray ionization tandem mass spectrometry using methods that have been described previously [92,93].

### Proteomic quantitation of LPP3

LPP3-derived tryptic peptides were quantitated using capillary flow reverse phase HPLC and electrospray ionization tandem mass spectrometry using an AB Sciex 5600 Q-TOF mass spectrometer (AB SCIEX, Framingham, MA) operated in high-resolution selected ion monitoring mode. We quantitated the following peptides STIQNPYVAALYK, NGGSPALNNNPR, EILSPVDIIDR and used a mass-labelled derivative of the latter of these (New England Peptides, Gardner, MA) as an internal standard [94].

### Immunohistochemistry

Immunohistochemical staining for LPP3 was undertaken using mouse monoclonal antibody clone 7H7D3 (Biosensis, Temecula, CA), which has previously been validated, including for immunohistochemistry [95]. Formalin-fixed paraffin-embedded tissue samples of non-atherosclerotic aorta (n = 5), and atherosclerotic aorta (n = 5) obtained with full ethical approval from the National Research and Ethics Service (Oxfordshire Research and Ethics Committee A: reference 04/Q1604/21), were immunostained using the Bond Max™ fully automated immunohistochemistry system (Leica Microsystems, Wetzlar, Germany). Heat induced antigen retrieval was performed using the EDTA-based Novocastra Bond Epitope Retrieval Solution 2 (pH 9.0) for 30 minutes and the NovolinkTM max polymer detection system (Leica Microsystems) was used, as per the manufacturer’s instructions. Detection reagents only were used as a negative control. Slides were mounted in Aquatex mounting medium (Merck, Darmstadt, Germany). Stained sections were photographed with a Nikon DS-FI1 camera with a Nikon DS-L2 control unit (Nikon, Kingston-upon-Thames, UK) and an Olympus BX40 microscope (Olympus, Watford, UK). All immunohistochemical staining was analyzed by and performed under the supervision of an experienced board certified consultant pathologist (ES).

## Supporting Information

S1 Fig Foam cell formation in human primary macrophages. Light microscopy of primary human macrophages (A, C) and oxLDL treated macrophages (B, D) stained with oil red O and visualized with (C, D) and without (A, B) phase contrast demonstrates extensive red-colored intracellular lipid in oxLDL-treated cells which confirms foam cell formation (scale bar indicates 100 um).
(EPS)

S2 Fig Genomic distribution of open chromatin sites in macrophages and foam cells. The images were annotated using CEAS and the background genome distribution derived from percentage of genome covered by RefSeq annotations.
(EPS)

S3 Fig Width of dynamic FAIRE sites. The frequency distributions of widths of dynamic chromatin sites suggest most are not more than one nucleosome in width with a relative increase in wider sites at promoters.
(EPS)

S4 Fig Up-regulation of *IL6R* and *PPAP2B* in foam cells compared to macrophages as assessed by quantitative RT-PCR. (A) OxLDL induces expression of IL6R at the mRNA level (n = 3). (B) OxLDL induces expression of *PPAP2B* at the mRNA level (n = 3, homozygous major genotype rs72664324, rs17114036).
(EPS)

S5 Fig EMSA with cold probe competition assays. Only the C/EBP consensus sequence competes off binding to the A allele. Cold probes for NFY, STAT5A and GR did not compete off binding. No protein binding to the G allele was detectable.
(EPS)

S6 Fig EMSA with probes for the rs72664324 alleles. The A allele binds C/EBP beta as demonstrated by a super-shift with anti-C/EBP beta antibody. The anti-C/EBP alpha antibody produces no super-shift.
(EPS)

S1 Table (A) List of differentially expressed genes (probe centric) between macrophages and foam cells using paired data (FDR adjusted p < 0.05). For some genes several different probes are reported. (B) List of differentially expressed genes (probe centric) without using pairing information (FDR adjusted p < 0.05). For some genes several different probes are reported. (XLSX)

S2 Table CAD loci and SNPs in high linkage disequilibrium. (XLSX)

S3 Table Genes within 50kb of CAD SNPs. (XLSX)

S4 Table Gene set enrichment analysis for genes within 50kb of CAD SNPs. (XLSX)

S5 Table Macrophage open chromatin sites identified by FAIRE-seq. (XLSX)

S6 Table Foam cell open chromatin sites identified by FAIRE-seq. (XLSX)

S7 Table Dynamic FAIRE-seq sites. (XLSX)

S8 Table Top 10 enriched transcription factor binding site motifs in dynamic open chromatin sites (top 3000 open dynamic chromatin sites, see also S9 Table). (PDF)

S9 Table Complete output of transcription factor motif enrichment analysis in dynamic chromatin sites (top 3000 open chromatin sites, see also S8 Table). (XLSX)

S10 Table Dynamic H3K27ac sites. (XLSX)

S11 Table Macrophage super enhancer clusters. (TXT)

S12 Table Foam cell super enhancer clusters. (TXT)

S13 Table Complete output of transcription factor motif enrichment analysis in the subset of 1743 dynamic chromatin sites with dynamic enhancer status. (XLSX)

S14 Table Dynamic C/EBP beta binding sites. (TXT)

S15 Table Transcription factor motif enrichment in dynamic C/EBP beta sites to confirm C/EBP beta motif enrichment (on top 3000 sites). (XLSX)

S16 Table Count of overlaps of CAD SNPs with chromatin and enhancer maps. (XLSX)

S17 Table Transcription factor motif affinities for rs72664324 alleles. (PDF)
