# Supplementary material for: Lipid-Induced Epigenomic Changes in Human Macrophages Identify a Coronary Artery Disease-Associated Variant that Regulates PPAP2B Expression through Altered C/EBP-Beta Binding
Source: PLoS Genet. 2015 Apr 2;11(4):e1005061. doi: 10.1371/journal.pgen.1005061 (PMC4383549; doi:10.1371/journal.pgen.1005061)
Supplement: S8 Table — (PDF) [file pgen.1005061.s014.pdf]

Rank	Matrix	Trnscription factor	DNA binding domain	Hits	P value (-10*log10)	Motif
1	MC00456	JUND	Leucine Zipper Family	1213	690.776	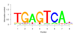
2	MS00288	TFE3	Helix-Loop-Helix Family	1569	690.776	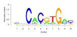
3	MC00339	NFE2L2	Leucine Zipper Family	1318	690.776	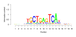
4	MC00319	EGR2	BetaBetaAlpha-zinc finger Family	2289	690.776	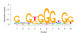
5	MA0067	PAX2	Homeodomain Family	1668	690.776	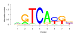
6	MC00163	Creb1	Leucine Zipper Family	1640	690.776	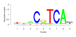
7	MC00337	Ikzf3	BetaBetaAlpha-zinc finger Family	976	688.093	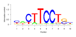
8	MS00302	CEBPG	Leucine Zipper Family	522	687.539	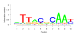
9	MS00223	SP3	BetaBetaAlpha-zinc finger Family	2377	671.922	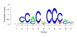
10	denovo16	EGR1	BetaBetaAlpha-zinc finger Family	2970	631.408	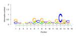
