# Supplementary material for: Lipid-Induced Epigenomic Changes in Human Macrophages Identify a Coronary Artery Disease-Associated Variant that Regulates PPAP2B Expression through Altered C/EBP-Beta Binding
Source: PLoS Genet. 2015 Apr 2;11(4):e1005061. doi: 10.1371/journal.pgen.1005061 (PMC4383549; doi:10.1371/journal.pgen.1005061)
Supplement: S17 Table — (PDF) [file pgen.1005061.s023.pdf]

Rank	P Value	Matrix ID	Matrix Name
Alternative A allele			
1	<0.000625	M00493	V$STAT5A_03
2	<0.000625	M00921	V$GR_Q6_01
3	<0.000625	M00500	V$STAT6_02
4	<0.000625	M00496	V$STAT1_03
5	<0.000625	M00498	V$STAT4_01
6	0.000625	M00494	V$STAT6_01
7	0.00853	M00747	V$IRF1_Q6
8	0.00932	M01250	V$E2F1_01
9	0.0102	M01147	V$DMRT2_01
10	0.0113	M00181	V$E2_Q6
11	0.0116	M00928	V$E2_Q6_01
12	0.0123	M00107	V$E2_01
13	0.0137	M00190	V$CEBP_Q2
14	0.0245	M01123	V$NANOG_01
15	0.0249	M01252	V$E2F6_01
16	0.0263	M00116	V$CEBPA_01
17	0.0282	M01281	V$NFAT1_Q6
18	0.036	M00185	V$NFY_Q6
19	0.0449	M00463	V$POU3F2_01
20	0.0452	M00621	V$CEBPDELTA_Q6
21	0.0471	M00309	V$ACAAT_B
22	0.0473	M00750	V$HMGIY_Q6
23	0.0498	M00775	V$NFY_Q6_01
Reference G allele			
1	<0.000938	M00493	V$STAT5A_03
2	<0.000938	M00921	V$GR_Q6_01
3	<0.000938	M00500	V$STAT6_02
4	<0.000938	M00496	V$STAT1_03
5	<0.000938	M00498	V$STAT4_01
6	0.000938	M00494	V$STAT6_01
7	0.00287	M00181	V$E2_Q6
8	0.0053	M00928	V$E2_Q6_01
9	0.00563	M00107	V$E2_01
10	0.00852	M00747	V$IRF1_Q6
11	0.00931	M01250	V$E2F1_01
12	0.0203	M01123	V$NANOG_01
13	0.0249	M01252	V$E2F6_01
14	0.0282	M01281	V$NFAT1_Q6
15	0.0314	M00792	V$SMAD_Q6
16	0.0443	M00070	V$TAL1BETAITF2_01
17	0.0475	M00750	V$HMGIY_Q6
